# Repeat physical activity measurement by accelerometry among colorectal cancer patients—feasibility and minimal number of days of monitoring

**DOI:** 10.1186/s13104-015-1168-y

**Published:** 2015-06-06

**Authors:** Stephanie Skender, Petra Schrotz-King, Jürgen Böhm, Clare Abbenhardt, Biljana Gigic, Jenny Chang-Claude, Erin M Siegel, Karen Steindorf, Cornelia M Ulrich

**Affiliations:** Division of Preventive Oncology, National Center for Tumor Diseases (NCT) and German Cancer Research Center (DKFZ), Heidelberg, Germany; Division of Clinical Epidemiology, German Cancer Research Center (DKFZ), Heidelberg, Germany; Department of Cancer Epidemiology, H. Lee Moffitt Cancer Center and Research Institute, Tampa, FL USA; Cancer Prevention Program, Fred Hutchinson Cancer Research Center, Seattle, WA USA; Huntsman Cancer Institute, Salt Lake City, UT USA

**Keywords:** Feasibility, Physical activity, Measurement, Colorectal cancer, Accelerometry

## Abstract

**Background:**

Physical activity plays an important role in colorectal cancer and accelerometry is more frequently used to measure physical activity. The aim of this study was to evaluate feasibility of physical activity measurement by accelerometry in colorectal cancer patients under free-living conditions at 6, 12 and 24 months after surgery, to evaluate the appropriate wear time and to compare results to pedometry.

**Methods:**

Colorectal cancer patients (stage 0/I–IV) from the ColoCare study were asked to optionally wear an accelerometer and a pedometer for ten consecutive days 6, 12 and 24 months post-surgery. Participants completed a feedback questionnaire about the accelerometer measurement. The course of moderate-to-vigorous physical activity over the 10 days was investigated. Additionally, daily step counts from accelerometers and pedometers were compared.

**Results:**

In total, there were 317 individual time points, at which 198 participants were asked to wear an accelerometer. Fifty-nine% initially agreed to participate and of these, 83% (n = 156) completed the assessment with at least 4 days of data. Twenty-one% more consents were obtained when participants were asked on a face-to-face basis compared to recruitment by telephone (P = 0.0002). There were no significant differences in time spent in moderate-to-vigorous physical activity between different wear-time lengths of accelerometry. Both Spearman and intraclass correlation coefficients showed strong correlations (0.92–0.99 and 0.84–0.99, respectively) of moderate-to-vigorous physical activity across 3, 4, 7 and 10 days measurement. Step counts measured by accelerometry and pedometry were strongly correlated (ρ = 0.91, P < 0.0001).

**Conclusion:**

This study suggest that accelerometry is a feasible method to assess physical activity in free-living colorectal cancer patients and that three valid days of physical activity measurement are sufficient for an accurate assessment.

**Electronic supplementary material:**

The online version of this article (doi:10.1186/s13104-015-1168-y) contains supplementary material, which is available to authorized users.

## Background

Physical activity plays an important role in colorectal cancer patients. A 30–60% improved colorectal cancer specific survival has been reported for patients with higher levels of activity [1–5]. Quality of life can also be positively affected by an active lifestyle [6]. The American Cancer Society Guidelines suggest that cancer survivors engage in at least moderate intensity physical activity for at least 150 min/week [7]. Nevertheless, to date it is not clear whether resistance or aerobic exercise is more beneficial for cancer survivors. In addition, not much is known about the effect of the timing, frequency and intensity on survival. This highlights the importance for investigations of the effects of physical activity in cancer patients.

Physical activity is defined as any bodily movement that results in expenditure of energy [8]. Assessment of total bodily movement in free-living conditions, however, is a challenge. Various methods exist to measure physical activity, including behavioral observations, questionnaires, physical activity diaries, direct/indirect calorimetry, and, more recently, motion sensors, such as accelerometers or pedometers. The most cost-effective method to measure physical activity is the administration of physical activity questionnaires which, however, provides subjective information that may over- or underestimate participants’ physical activity behaviors [9, 10]. In particular, older adults are more likely to engage in light- to moderate-intensity physical activity, which is the most difficult type of activity to assess by questionnaire [11]. As an objective and non-invasive device, motion sensors, such as pedometers or accelerometers are increasingly implemented in epidemiological studies. Pedometers were designed to count the number of steps of the person wearing it. They are easy to use and often function as a motivational tool [12]. Accelerometers are small electronic devices that record acceleration of change of body movement and provide an objective estimate of duration and intensity of locomotion [13]. For objective assessment of physical activity in free-living conditions, accelerometers are typically worn for several consecutive days during waking hours. However, wearing such devices may lead to motivational bias, probably due to the participants knowing that their physical activity habits are observed [14].

In colorectal cancer patients, attachment of the devices around the hip or the waist might interfere with surgical wounds or scars, or compliance to wearing the devices might be influenced by side-effects of chemo- or radiotherapy. Currently, there are only few studies investigating physical activity with accelerometry in colorectal cancer patients [15–19]. Not much is known about the feasibility of physical activity assessment by accelerometry and appropriate wear times. The objective of this manuscript is to assess the acceptance of accelerometry measurement among colorectal cancer patients, to determine the appropriate duration and to compare step counts from pedometry and accelerometry. The key aspects of these investigations is to reduce the burdens that are associated with wearing accelerometers in colorectal cancer patients, in order to achieve the best possible compliance, concurrently with an accurate assessment.

## Methods

### Study population

This study is nested in the prospective ColoCare study, an international cohort of newly diagnosed stage I–IV colorectal cancer patients (ICD-10 C18-C20). The ColoCare Consortium is a multicenter initiative of interdisciplinary research on colorectal cancer outcome and prognosis, and comprises patient recruitment at the National Center for Tumor Diseases, Heidelberg (Germany), the Fred Hutchinson Cancer Research Center, Seattle (WA, USA) and the H. Lee Moffitt Cancer Center and Research Institute, Tampa (FL, USA). All study centers follow the same standard operating procedures (SOPs) with respect to patient recruitment, biospecimen handling, patient follow-up and medical data abstraction. ColoCare inclusion criteria are: first-diagnosed colon or rectal cancer (stages I–IV), age >18 years, English (US sites) or German (German site)-speaking, and mentally/physically able to consent and participate. Subjects meeting the inclusion criteria are recruited to the ColoCare study prior to tumor surgery. Baseline examination includes anthropometric measurements, biospecimen collection (blood, urine, feces, and fresh frozen tissue), and self-administered questionnaires on symptoms and health-related quality-of-life. Participants are followed-up (1) passively by retrieving medical data from hospital records, and (2) actively at 3, 6, 12, 24 and 36 months post-surgery with collection of blood, stool, urine, questionnaires on symptoms, health-related quality-of-life, and dietary assessment by food frequency questionnaire.

Physical activity was assessed by accelerometry and pedometry at the Heidelberg site. Accelerometers and pedometers were offered to participants as an optional study assessment at 6, 12 and 24 months post-diagnosis. During the initial phase of the physical activity assessment, from September 2011 to July 2012, only participants who came to the study office for their follow-up visits were asked to wear a device and from July 2012, all participants at each follow-up time point were asked, either in person if they came to the National Center for Tumor Diseases (NCT) Heidelberg or via telephone if we did not see them in person because their follow-up was conducted at their local general practitioners. The last chemotherapy cycle had to be finished at least 2 weeks before each individual follow-up visit.

The assessment of physical activity was offered as an optional component at study outset per study design. The present study included n = 156 physical activity assessments from 102 patients of the ColoCare study site in Heidelberg, because some participants took part multiple times at their different follow-up time points. The study was approved by the Institutional Review Boards in Heidelberg and all study participants provided informed consent.

### Data collection

The Actigraph GT3x+ (Actigraph, Pensacola, FL) accelerometers are small, light-weight devices that assess physical activity in three axes. It can record raw acceleration data, activity counts, step counts, energy expenditure, amount of sleep, and has an integrated light sensor. This device was attached on an elastic belt and patients were instructed to wear it below their chests. The chest was chosen as attachment site in order not to interfere with the surgical scars or stomas. Data was collected in 30 Hz intervals. Raw data was downloaded and processed using the ActiLife software (ActiGraph, Pensacola, FL, version 6.6.3). Accelerometer data was then summed up into 10 s epochs. Data were considered valid if the devices were worn for at least 4 days and for at least 6 h per day. Non-wear time was defined as at least 60 min of consecutive zero counts with a 2 min interruption tolerance [20]. Cut-points for the different physical activity levels were defined as follows: light activity (100–1,951 counts per minute), moderate activity (1,952–5,724 counts per minute), vigorous activity (≥5,725–20,000 counts per minute) and moderate-to-vigorous activity (≥1,952–20,000 counts per minute) [21]. Accelerometry data exceeding 20,000 counts per minute were considered spuriously high data and thus, excluded from analyses [20]. The Omron Walking Style Pro (HJ 720 IT, Omron, Japan) is a small pedometer device with a memory capacity of 41 days. It can be attached on the hip with a clip and comes with compatible software (Health Management Software, Omron, Japan). Those days considered as non-wear time for accelerometry were also excluded for pedometry. Participants wore both devices for ten consecutive days, only during waking hours but not during water-based activities. Following the 10 day physical activity assessment, participants filled out a feedback questionnaire with three questions concerning the acceptance of the devices. Participants were given post-paid parcels in order to return the devices along with information about the measurement, an informed consent, written instructions, a wear-diary and the feedback questionnaire. After data were downloaded and evaluated, each participant received a personal summary of their measured physical activity data.

Reasons for refusals and drop outs were assessed during the follow-up visits or phone calls with the study participants and recorded in the database.

### Data analysis

Accelerometry data is presented as steps per day and minutes in moderate-to-vigorous physical activity (MVPA) per day. For minutes in moderate-to-vigorous physical activity per week, total amount of moderate-to-vigorous physical activity was divided by the number of measurement days and then multiplied by 7. For stratified analyses investigating differences by patient characteristics, age was dichotomized at 65 years and BMI at 25 kg/m^2^. Tumor stages were grouped as stages 0–II and stages III–IV. Descriptive methods were used to present information about the study population and participation numbers. To investigate if there were differences in participation and drop-outs by follow-up location, age groups, sex, BMI groups or stage groups, as well as differences in tumor stages between refusals and completed assessments we performed χ^2^ tests.

We assessed physical activity by accelerometry at three different time points to investigate the feasibility in colorectal cancer patients. In order to investigate if participants who reported to be sometimes more motivated to engage in physical activity due to the measurement actually were more active, we performed a Student T Test, using the square-root transformed MVPA variable. We performed a Wilcoxon signed rank test in order to investigate if there were differences in MVPA minutes between weekdays or weekend days. To analyze differences between defined time periods within the 10 days measurement, individual consecutive days were grouped into four ways: days 1–3, days 1–7, days 4–10 and days 8–10, which is equivalent to the first 3 and 7 days and the last 7 and 3 days, respectively. For comparison of times spent in sedentary, light and moderate-to-vigorous physical activities in these four groups, Wilcoxon signed rank tests were performed. The intraclass correlation coefficient (ICC) was estimated in order to assess the absolute agreement between different measurement time periods (first 3, 4, 7, 10 days). Additionally, the Spearman correlation coefficient (ρ) was estimated to investigate if participants ranked similarly across the different time periods. Strong correlation was considered at 0.80. For all analyses statistical significance was reached at the p < 0.05 level.

The Spearman correlation coefficient (ρ) was determined between step counts measured by accelerometer and pedometer. Additionally, a linear regression with accelerometer step and pedometer step counts was performed to predict step counts from pedometry by step counts from accelerometry. Statistical analyses were performed using SAS (version 9.3).

## Results

At 317 follow-up time points, a total of 198 participants were offered to wear an accelerometer for ten consecutive days. Table 1 displays the characteristics of patients who refused and of those who actually participated, by follow-up time point. There were 13 participants who completed measurements at all three time points. Out of 40 participants who completed the 6 month assessment 31 (78%) also completed the 12 month follow-up measurement. Participants who refused the accelerometer assessment at the 6 month time point were significantly older than those who completed the measurement with at least four valid days (P < 0.05). However, we did not observe any statistically significant associations between any of the other variables at all time points.

**Table 1:** Characteristics of participants who refused and who completed by follow-up time point

	6 months follow-up		12 months follow-up		24 months follow-up	
	Refused	Completed	Refused	Completed	Refused	Completed
N^a^ (times asked)	22 (18%)	65 (54%)	38 (31%)	58 (48%)	29 (40%)	33 (46%)
Sex						
Male	17 (77%)	40 (62%)	24 (63%)	40 (69%)	17 (59%)	21 (64%)
Female	5 (23%)	25 (38%)	14 (37%)	18 (31%)	12 (41%)	12 (36%)
Age, years (mean, SD)	67.3 (12.9)	60.7 (13.4)	64.0 (11.4)	61.2 (11.1)	63.5 (11.7)	63.0 (10.9)
BMI kg/m^2^ (mean, SD)	24.4 (4.8)	25.6 (3.6)	26.5 (3.9)	26.1 (3.3)	26.6 (4.6)	27.5 (4.3)
Tumor stage						
0 + I	6 (27%)	9 (14%)	12 (32%)	14 (24%)	11 (38%)	6 (18%)
II	4 (18%)	19 (29%)	11 (29%)	18 (31%)	6 (21%)	14 (42%)
III	7 (32%)	21 (31%)	9 (24%)	15 (26%)	9 (31%)	11 (33%)
IV	4 (18%)	7 (11%)	6 (16%)	7 (12%)	3 (10%)	1 (3%)
Not known	1 (5%)	9 (15%)	0	4 (7%)	0	1 (3%)
Participation in accelerometry at prior follow-up						
6 months	n.a.		40	31 (78%)	25	14 (56%)
12 months	n.a.		n.a.	n.a.	32	20 (63%)
6 and 12 months	n.a.		n.a.	n.a.	19	13 (68%)

Thirty-eight participants took part in the optional physical activity assessment multiple times at the different follow-up time points, whereas 64 participants wore the devices just once.

*N* number of follow-up approaches, *n.a.* not applicable, *kg* kilogram, *m* ^2^ square meter, *SD* standard deviation.

^a^Out of those participants who were asked.

Information on participation and refusals is shown in Additional file 1. Of the 317 times where patients were asked whether they were interested to participate in the assessment of their physical activity, 59% initially gave their written informed consent and 28% refused. Twelve% of the participants, who gave their informed consent, dropped out and 156 (83%) measurements were completed with at least 4 days. Of the 156 completed measurements 72% were completed with at least 10 days and 95% with at least 7 days of data. The percentage of obtained informed consents was 21% higher when participants were asked on a face-to-face basis compared to recruitment by telephone (P = 0.0002). No statistical significant differences could be observed when comparing participation or drop outs between men and women, age, BMI, follow-up location and stage group within the three follow-up time points.

Thirty-eight participants took part in the optional physical activity assessment multiple times at different follow-up time points, whereas 64 participants wore the devices just once, because they were not scheduled for other follow-up time points.

Main reasons for refusals were that participants felt that wearing the accelerometers and pedometers for 10 days was too much of a burden for them (n = 12) or they thought it would be uncomfortable and that the devices would annoy them during their leisure time activities or during work (n = 10). Other reasons for refusals were paused follow-ups (n = 4), psychologically not being able to participate (n = 4), participants did not want to be reminded of the disease (n = 4), recent or upcoming surgery (n = 3), bad general health status (n = 1), or being too hot outside (n = 1). At 44 refusals no information was available. Reasons for drop-outs were bad general health status (n = 4), chemotherapy side-effects (n = 2), inconvenience (n = 5), depleted batteries due to unarranged postponements (n = 2), or being too hot outside (n = 1). No information was available for ten drop-outs. Fifty-eight percent of the drop-outs did not wear the devices at all and 21% wore the accelerometers for 3 days.

Based on the feedback questionnaire, 84% of patients reported that they felt just a little or not at all uncomfortable wearing the cheststrap (Table 2). When being asked about additional motivation for physical activity during the wear-period 115 times (74%) patients noted that they were not motivated to be more active. Fifteen% reported they were sometimes more motivated to be more active, however no statistically significant differences could be observed when comparing these participants to those who did not report that they were sometimes more motivated. This feedback did not differ across the three time points.

**Table 2:** Results from the feedback questionnaire

	6 months	12 months	24 months	Total
Did you feel uncomfortable wearing the chest strap with sensor?				
Not at all	36 (51%)	33 (52%)	13 (65%)	82 (53%)
A little	23 (33%)	20 (31%)	5 (25%)	48 (31%)
Quite a bit	5 (7%)	9 (14%)	2 (10%)	16 (10%)
Very much	6 (9%)	2 (3%)	0	9 (6%)
Was it difficult to remember wearing the chest strap every day?				
No	67 (96%)	61 (95%)	19 (95%)	147 (95%)
Yes	3 (4%)	3 (5%)	1 (5%)	7 (5%)
Did wearing the chest strap motivate you to be more active than usual?				
No	53 (76%)	47 (73%)	15 (75%)	115 (75%)
Yes, in the beginning	6 (9%)	5 (8%)	3 (15%)	14 (9%)
Sometimes	9 (13%)	12 (19%)	2 (10%)	23 (15%)
Yes, all of the time	2 (3%)	0	0	2 (1%)

**Figure 1:**
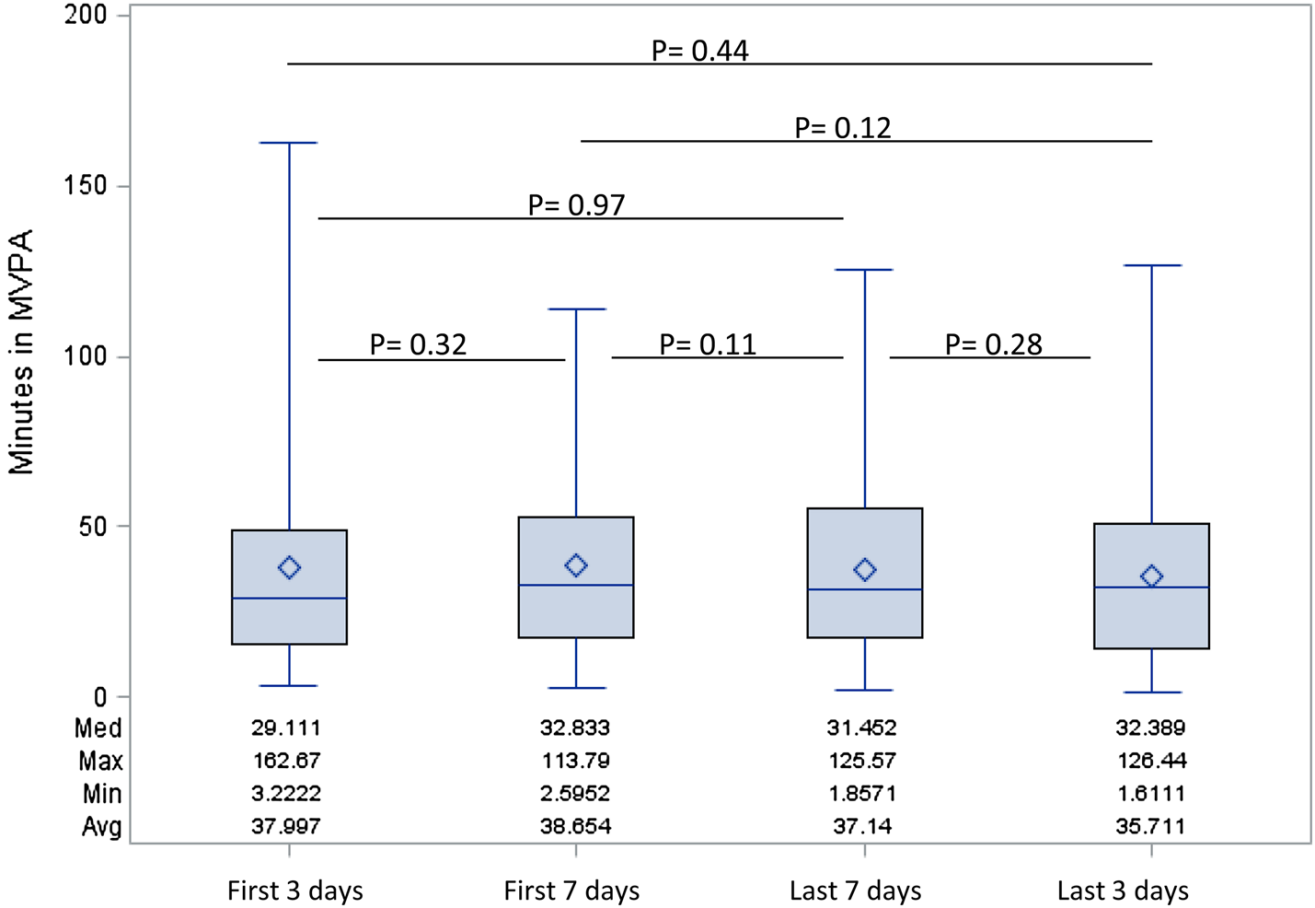
Comparison of physical activity measurement using different wear-time lengths of accelerometry. Minutes in moderate-to-vigorous physical activity within the first 3 and 7 days and the last 3 and 7 days.

No significant differences were observed between weekdays and weekend days with mean differences of 0.1 min (P = 0.08) at 6 months, 3.1 min (P = 0.08) at 12 months and −0.1 min (P = 0.42) at 24 months post-surgery. Accelerometer-measured time in moderate-to-vigorous physical activity for the grouped days is shown in Figure 1. When comparing average physical activity times by grouped days (first 3, first 7, last 3, last 7), no significant differences in time spent in moderate-to-vigorous physical activity were observed. Additionally, we did not observe significant differences in light or sedentary physical activity across the different wear-time periods.

The intraclass correlation coefficient showed very strong agreement for MVPA across the different measurement time periods (first 3, 4, 7, 10 days) ranging from ICC = 0.84 [95% confidence interval (CI) 0.75, 0.90] to ICC = 0.99 (95% CI 0.98, 0.99) **(**Table 3). The Spearman correlation coefficient also showed strong correlations of moderate-to-vigorous physical activity across the varying measurement lengths (first 3, 4, 7 and 10 days) at all time points ranging from ρ = 0.92 (P < 0.0001) to 0.99 (P < 0.0001) (Table 3). Additional file 2 depicts the association between minutes spent in moderate-to-vigorous physical activity within the first 3 days and the entire 10 days at all three time points. Even though participants spent in average about 6–9 min more time on moderate-to-vigorous physical activity during the first 3 days at 6, 12 and 24 months, the ranking of the participants based on the first 3 vs. overall 10 days is very consistent.

**Table 3:** Spearman correlation coefficients and intraclass correlation coefficients between different measurement periods

	First 4 days ICC, (95% CI) ρ, p value*	First 7 days ICC, (95% CI) ρ, p value*	10 days ICC, (95% CI) ρ, p value*
6 months			
First 3 days	0.98, (0.97, 0.99) 0.98*	0.90, (0.84, 0.94) 0.95*	0.84, (0.75, 0.90) 0.95*
First 4 days		0.93, (0.89, 0.96) 0.95*	0.88, (0.81, 0.93) 0.95*
First 7 days			0.98, (0.97, 0.99) 0.99*
12 months			
First 3 days	0.99, (0.98, 0.99) 0.98*	0.94, (0.90, 0.96) 0.95*	0.90, (0.84, 0.94) 0.92*
First 4 days		0.96, (0.93, 0.98) 0.96*	0.93, (0.86, 0.96) 0.94*
First 7 days			0.99, (0.98, 0.99) 0.99*
24 months			
First 3 days	0.96, (0.92, 0.98) 0.96*	0.93, (0.87, 0.96) 0.92*	0.93, (0.87, 0.96) 0.92*
First 4 days		0.96, (0.92, 0.98) 0.96*	0.94, (0.88, 0.97) 0.96*
First 7 days			0.98, (0.96, 0.99) 0.99*

*ICC* intraclass correlation coefficient, *CI* confidence interval, *ρ* Spearman’s correlation coefficient.

* P < 0.0001.

Median step counts measured by pedometers and accelerometers were 4,654 steps and 5,301 steps, respectively. Step counts measured by the two devices were strongly correlated (ρ = 0.91, P < 0.0001). Pedometer step counts are, in general, predicted to be lower than accelerometer step counts.

## Discussion

In this study we investigated the feasibility of physical activity assessment by accelerometry in a colorectal cancer patient cohort with repeat assessments over time, in the context of days of wear-time, follow-up site (face-to-face at the NCT versus telephone), follow-up time point, sex, age, BMI and stage. We conclude that accelerometry is a feasible method to assess physical activity in colorectal cancer patients independent of gender, tumor stage or BMI, as 83% of the physical activity measurements were completed with data from at least four consecutive days. In addition, our study suggests that an assessment of 3 or 4 days is sufficient for the assessment of physical activity of our study participants.

Maddocks et al. [14] even reported a compliance of 98% in their study with lung and gastrointestinal cancer patients recruited from oncology clinics. Our compliance was lower because of the fact that the physical activity assessment was an optional part of the main study with extensive data collection. Additionally, our study participants wore the device below their chest, whereas Maddocks’ et al. participants wore the devices on their inner thigh. In a study with advanced colorectal cancer patients who were asked to wear an accelerometer for 72 continuous hours attached on the wrist a compliance of 68% was reported [18].

As expected, approaching participants on a face-to-face basis led to a higher number in participation and a lower number in refusals compared to telephone recruitment. There were no significant differences in participation, refusal or drop-outs between any of the patient characteristics investigated (age, sex, BMI, follow-up site or stage). Concerning sex and BMI, our results are in agreement with the results from Roth et al. [22] who did not observe any statistically significant differences in participation, refusal or non-participation (<4 days of valid data) in waist-worn accelerometry measurement. However, with regards to age, they reported that non-participants were younger compared to those who completed the measurements with at least 4 days. Hassani et al. [23] investigated the association of different factors with non-consent to a wrist-worn accelerometer. They did not observe any association with age but they reported that women were more likely to refuse. However, we need to be cautious when comparing these data, as each of the three studies defined their attachment site differently (chest vs. waist vs. wrist).

Wearing physical activity monitors may lead to promotion of physical activity due to increased personal motivation by the participants as well as the awareness of their activity levels being “observed”. Therefore, participants might be more active in the beginning of the measurement with this effect slowly leveling off with wear time. This was observed in a study by Maddocks et al. [14] in patients with lung or upper gastrointestinal cancer. Despite the fact that in 15% of the feedback questionnaires participants reported that they were sometimes motivated to be more active, we observed minimal differences when comparing the first 3 days to the last 3 days or the entire 10 days.

There were no differences in moderate-to-vigorous physical activity (minute) between the first 3 and the last 3 days within the entire 10 days measurements. Although statistically significant by Wilcoxon Signed Rank Test, differences between the first 7 vs 10 days were not meaningful (1.03 min). Additionally, in our study there were negligible differences in the ranking of participants if only the first 3, 4, 7 or 10 days were used for analyses. Matthews et al. [24] suggest that 7 days are a sufficiently long timespan to cover weekdays and weekend days and to achieve intraclass correlations of more than 80% in most populations. Our results suggest that perhaps even three valid days of physical activity measurement in our study population are sufficient as we achieved ICCs between 0.84 and 0.93 when comparing the first 3 days with the entire 10 days at all three follow-up time points. This is also in agreement with results from previous studies, suggesting that 3–7 days or even 3–5 days should be sufficient to assess physical activities in daily life [25, 26]. To date, as Broderick et al. [27] noted in a review article, there is no set standard for the required number of valid days. Some investigations suggest a minimum of six valid measurement days [14], whereas others suggest a minimum of two valid days [28].

Pedometers and accelerometers are more frequently used to objectively assess physical activity in studies with large numbers of participants. Pedometers, however, only count steps per day and are not able to identify intensities. Nevertheless, they are cheap and easy to apply in large scale studies. Pedometer and accelerometer step counts were highly correlated (ρ = 0.91, P < 0.0001). Medians of step counts assessed by accelerometers and pedometers differed by about 650 steps per day.

Similar to findings by Barreira [29], our results suggest that pedometer and accelerometer do not estimate comparable steps per day in colorectal cancer patients. This discrepancy could be explained by the different attachment sites of the devices in this study (chest vs. hip). Pedometers were attached on participants’ clothes, thus, participants frequently mentioned that they forgot to reattach the device after changing of clothes, or that they removed the pedometer during situations where formal clothing was required. These aspects led to differences in wear time and therefore to different values of step counts.

A strength of this study is the fact that we measured physical activity twice within the first year after surgery. Furthermore, the number of ten measurement days that were used in this study and the fact that each measurement was started on a Monday, Tuesday or Wednesday enabled us to investigate an entire week and in addition to compare the first 3 with the last 3 days which, consequently, were always the same weekdays. Information about weekdays and weekend days was not taken into account for the assessment of appropriate wear time, because our analyses showed no significant differences in the level of physical activity between weekdays and weekend days.

The limitations of single-mounted physical activity assessment devices are that they are not able to measure stationary movement, such as strength training or cycling. Additionally, they can only assess physical activity from the timeframe they were worn and pedometers can only report step counts and do not give information about the intensities of physical activity. Thus, none of these two methods is perfect, but since the measurement of step counts by the two devices showed similar results and accelerometers provide additional information compared to pedometers, pedometers could be omitted in this study.

Especially in diseased populations, wear-position should be carefully considered as it could negatively affect compliance and lead to drop outs due to pain or participants feeling uncomfortable with the attachment site. This is why we positioned the accelerometers below the chest even though, for the most accurate assessment of physical activity, the attachment close to the body’s center of mass is advised. According to a study by Cleland et al. [28] comparing data from multiple attachment sites, the differences in accuracy between the different sites were, although significant, reasonably low and therefore not highly relevant in practice. Positioning of activity monitors is an important aspect that should be considered especially in cancer patients.

## Conclusion

In conclusion, our results demonstrate that accelerometry is a feasible method to assess physical activity in free-living colorectal cancer patients with the device being attached below participants’ chests. In addition, only 3–4 days of wear time appear sufficient for accurate assessment and ranking of participants, which is an important finding in the context of reducing participant burden and cost.

## Additional files

**Additional file 1:** Participation numbers by time point, follow-up site, age, sex, BMI and stage.
**Additional file 2:** Comparison of physical activity measurement using two different wear time lengths of accelerometry and comparison of step counts with pedometry. a) Scatter plot of moderate-to-vigorous physical activity in first 3 days vs. 10 days b) Linear regression of accelerometry-based step counts with pedometry-based step counts with accelerometer steps as the dependent variable.

## Abbreviations

BMI: body mass index
ICC: intraclass correlation coefficient
ICD: International Statistical Classification of Disease and Related Health Problems
MVPA: moderate-to-vigorous physical activity
NCT: National Center for Tumor Diseases
