# Supplementary material for: Repeat physical activity measurement by accelerometry among colorectal cancer patients—feasibility and minimal number of days of monitoring
Source: BMC Res Notes. 2015 Jun 6;8:222. doi: 10.1186/s13104-015-1168-y (PMC4456792; doi:10.1186/s13104-015-1168-y)
Supplement: Supplementary file 1 — Additional file 1: Participation numbers by time point, follow-up site, age, sex, BMI and stage. [file 13104_2015_1168_MOESM1_ESM.pdf]

<u>Additional file 1.</u> Participation numbers by time point, follow-up site, age, sex, BMI and stage.

		IC obtained<br>N	Refused<br>N	Completed<br>N<br>(of IC obtained)	Drop out<br>N<br>(of IC obtained)
**Total** (n=317)		187 (59 %)	89 (28%)	156 (83 %)	22 (12 %)
**6M Follow-up** (n=121)		81 (67%)	22 (18%)	65 (80%)	12 (15%)
**12M Follow-up** (n=124)		68 (55%)	38 (31%)	58 (85%)	9 (13%)
**24M Follow-up** (n=72)		38 (53%)	29 (40%)	33 (87%)	1 (3%)
**Follow-up site**	On campus (n=190)	128 (67%)	40 (21%)	105 (82%)	16 (13%)
	Off-campus (n=127)	59 (46%)	49 (39%)	51 (86%)	6 (10%)
**Age**, years	< 65 (n=173)	109 (63%)	41 (24%)	92 (84%)	12 (11%)
	≥ 65 (n=144)	78 (54%)	48 (33%)	64 (82%)	10 (13%)
**Sex**	Male (n=209)	125 (60%)	58 (28%)	101 (81%)	16 (13%)
	Female (n=108)	62 (57%)	31 (29%)	55 (89%)	6 (10%)
**BMI,** kg/m²	<25 (n=107)	71 (66%)	25 (23%)	62 (87%)	7 (10%)
	≥25 (n=162)	112 (69%)	37 (23%)	93 (83%)	13 (12%)
**Stage**	0, I, II (n=168)	95 (57%)	50 (30%)	80 (84%)	12 (13%)
	III, IV (n= 132)	77 (58%)	37 (28%)	62 (81%)	10 (13%)

Abbreviations: n= numbers of follow-up approaches, IC, informed consent; M, month; BMI, body mass index; IC obtained: including number of drop outs; numbers and percentages within each row (IC obtained + refused) might not always sum up to the total n and 100% due to missing information about BMI: n=48; stage: n=41 ineligible patients (not shown in table); IC obtained= number of completed measurements+ number of drop outs+ number of devices lost
