# Supplementary material for: Repeat physical activity measurement by accelerometry among colorectal cancer patients—feasibility and minimal number of days of monitoring
Source: BMC Res Notes. 2015 Jun 6;8:222. doi: 10.1186/s13104-015-1168-y (PMC4456792; doi:10.1186/s13104-015-1168-y)

a) Scatter plot of moderate–to-vigorous physical (MVPA) activity in first 3 vs. 10 days

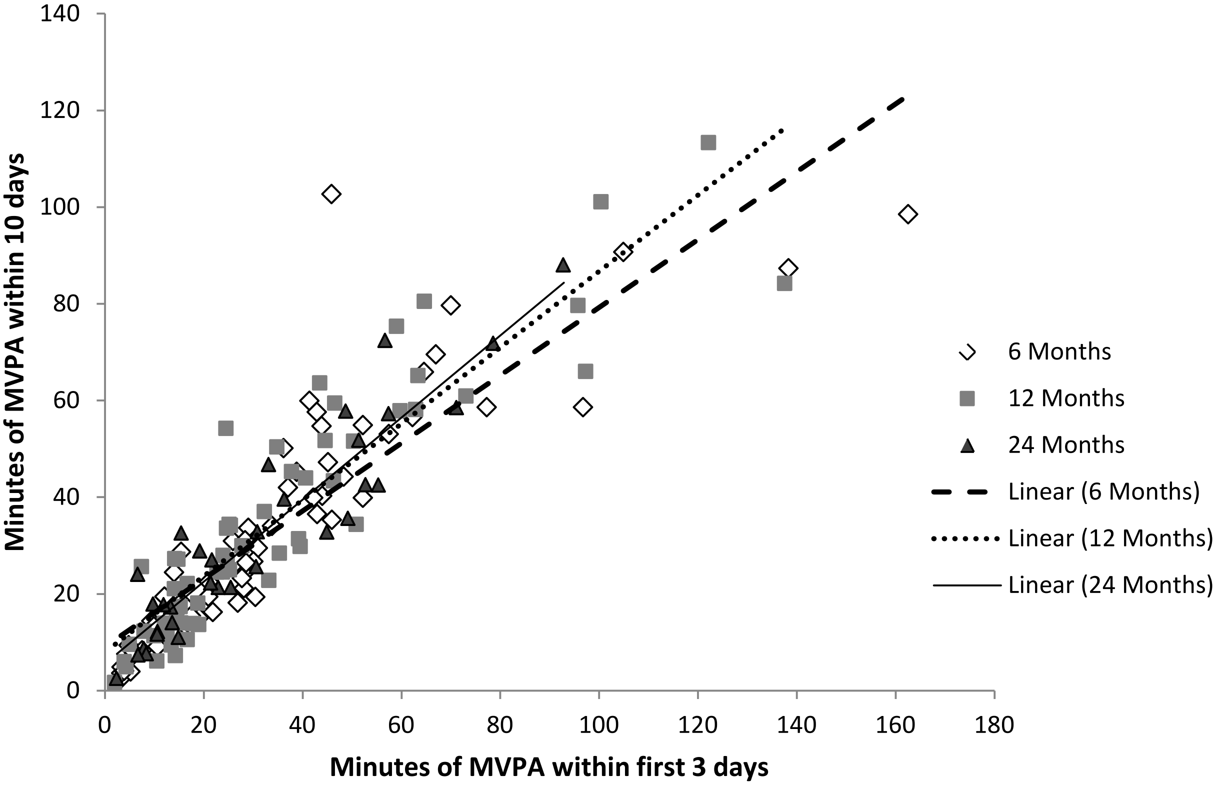

b) Linear regression of accelerometry-based step counts with pedometry-based step counts with accelerometer steps as the dependent variable

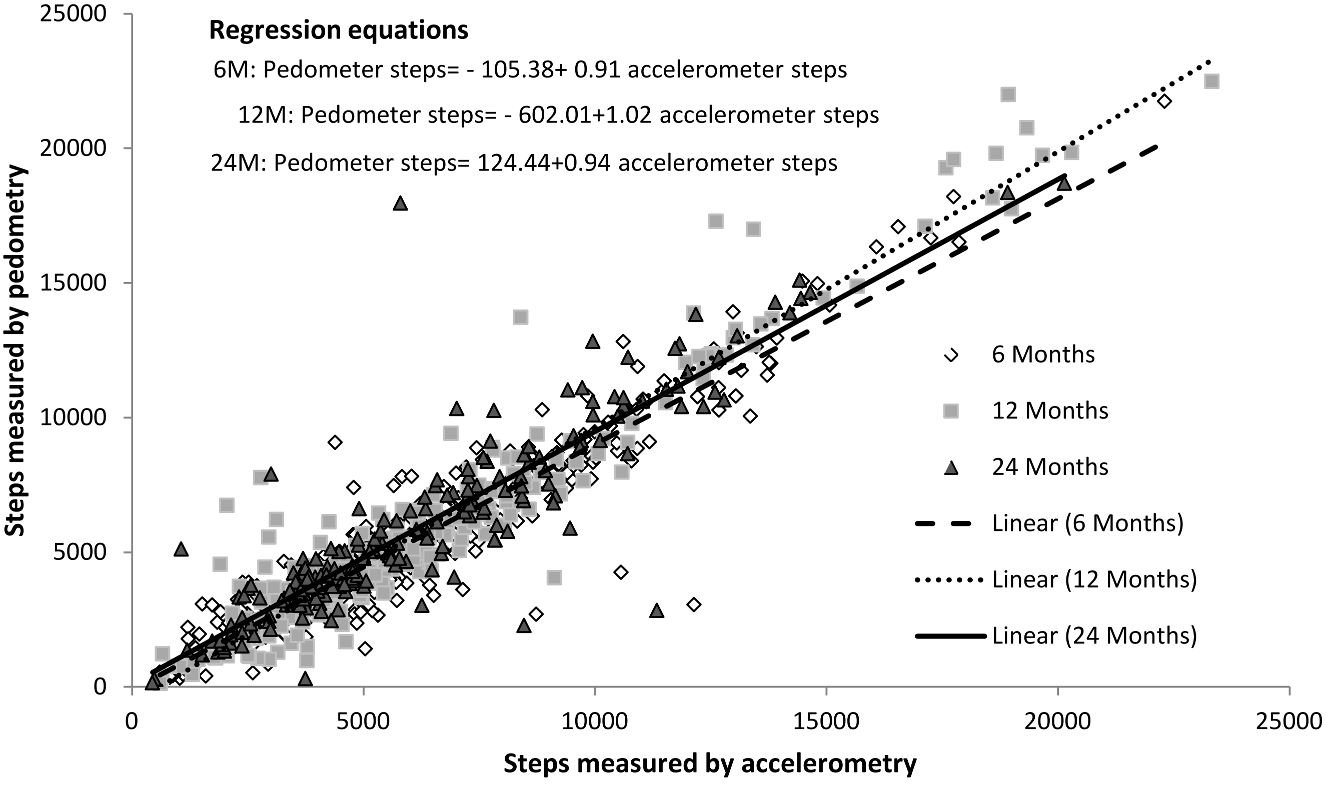

Supplement: Supplementary file 2 — Additional file 2: Comparison of physical activity measurement using two different wear time lengths of accelerometry and comparison of step counts with pedometry. a) Scatter plot of moderate-to-vigorous physical activity in first 3 days vs. 10 days b) Linear regression of accelerometry-based step counts with pedometry-based step counts with accelerometer steps as the dependent variable. [file 13104_2015_1168_MOESM2_ESM.pdf]
